# HEALTH COACHING FOR DEMENTIA CAREGIVERS: LESSONS LEARNED FROM THE ICARE4ME STUDY

**DOI:** 10.1093/geroni/igac059.2050

**Published:** 2022-12-20

**Authors:** Lauren Fisher, Lauren Massimo, Karen Hirschman

**Affiliations:** University of Pennsylvania, Philadelphia, Pennsylvania, United States; University of Pennsylvania, Philadelphia, Pennsylvania, United States; University of Pennsylvania, Philadelphia, Pennsylvania, United States

## Abstract

Frontotemporal dementia (FTD) is a common cause of young-onset neurodegenerative disease that causes progressive changes in behavior and personality. FTD is often diagnosed around age 60, creating complex care needs that result in caregivers of persons with FTD experiencing high rates of depression, burden, and poor self-care. The iCare4Me for FTD study (NCT04686266) randomized 15 caregivers to receive a virtual health coaching intervention over 6 months (10 sessions) and 15 caregivers to the control group. To better understand the caregivers’ experience with the health coach intervention, two focus groups with intervention group caregivers (n=5) were held. Focus groups were recorded, transcribed and coded using content analysis. Caregivers reported the most valuable aspect was the relationship that was developed with their health coach. Caregivers particularly valued having someone to talk to who was outside their immediate social and support networks. It was noted the structured self-care curriculum served as a good backbone for discussions, but more specific coping conversations related to loss of patient empathy, prognostic uncertainty, and anticipatory grief are needed. One caregiver described being, “… awash in grief and it's affected my memory” while another described “grief is a big issue and I don't really find too many people understand it, because my husband is alive, but so many parts of him are gone”. These findings will be used to inform future studies utilizing health coaches for caregivers of persons with FTD. Implications for evidence-based virtual health coaching interventions with caregivers of persons living with FTD will be described.

